# Thrombospondin-4 reduces binding affinity of [^3^H]-gabapentin to calcium-channel α_2_δ-1-subunit but does not interact with α_2_δ-1 on the cell-surface when co-expressed

**DOI:** 10.1038/srep24531

**Published:** 2016-04-14

**Authors:** Beatrice Lana, Karen M. Page, Ivan Kadurin, Shuxian Ho, Manuela Nieto-Rostro, Annette C. Dolphin

**Affiliations:** 1 Department of Neuroscience, Physiology and Pharmacology, University College London, Gower St., London WC1E 6BT, United Kingdom

## Abstract

The α_2_δ proteins are auxiliary subunits of voltage-gated calcium channels, and influence their trafficking and biophysical properties. The α_2_δ ligand gabapentin interacts with α_2_δ-1, and inhibits calcium channel trafficking. However, α_2_-1 has also been proposed to play a synaptogenic role, independent of calcium channel function. In this regard, α_2_δ-1 was identified as a ligand of thrombospondins, with the interaction involving the thrombospondin synaptogenic domain and the α_2_δ-1 von-Willebrand-factor domain. Co-immunoprecipitation between α_2_δ-1 and the synaptogenic domain of thrombospondin-2 was prevented by gabapentin. We therefore examined whether interaction of thrombospondin with α_2_δ-1 might reciprocally influence ^3^H-gabapentin binding. We concentrated on thrombospondin-4, because, like α_2_δ-1, it is upregulated in neuropathic pain models. We found that in membranes from cells co-transfected with α_2_δ-1 and thrombospondin-4, there was a Mg^2+^ -dependent reduction in affinity of ^3^H-gabapentin binding to α_2_δ-1. This effect was lost for α_2_δ-1 with mutations in the von-Willebrand-factor-A domain. However, the effect on ^3^H-gabapentin binding was not reproduced by the synaptogenic EGF-domain of thrombospondin-4. Partial co-immunoprecipitation could be demonstrated between thrombospondin-4 and α_2_δ-1 when co-transfected, but there was no co-immunoprecipitation with thrombospondin-4-EGF domain. Furthermore, we could not detect any association between these two proteins on the cell-surface, indicating the demonstrated interaction occurs intracellularly.

Ca_V_1 and Ca_V_2 voltage-gated calcium channels are associated with auxiliary β and α_2_δ subunits, which influence both the expression on the plasma membrane and the biophysical properties of the channels (for review see^1^,^2^). Understanding the mechanism of action of the α_2_δ-1 subunit is of translational importance, as it is the therapeutic target of the gabapentinoid drugs gabapentin and pregabalin^3^. These drugs were developed as antiepileptic agents, but also show efficacy in the treatment of neuropathic pain conditions^1^,^3^,^4^,^5^. We have found that these drugs reduce calcium currents chronically but not acutely, by inhibiting α_2_δ-1 and α_2_δ-2 trafficking^6^,^7^,^8^,^9^.

We have recently demonstrated that α_2_δ-1 and Ca_V_2.2 interact both intracellularly and at the plasma membrane, when these proteins are co-expressed^9^. In this and other studies, we found that the von Willebrand Factor-A (VWA) domain of α_2_δ subunits is important, both for cell surface expression of α_2_δ-1, and for mediating the enhancement by α_2_δ-1 of Ca_V_2 channel cell surface expression and function^9^,^10^,^11^. Structural evidence indicates that the region of interaction between α_2_δ-1 and Ca_V_1.1 involves the VWA domain as well as other regions of α_2_δ-1^12^. However the VWA domain may also interact with other protein(s) involved in calcium channel trafficking pathways.

The thrombospondins (TSPs) are multi-domain secreted extracellular matrix proteins (Fig. 1A) with diverse functions^13^, one of which is synaptogenesis^14^. TSPs are secreted from astrocytes and promote the formation of silent synapses, without postsynaptic receptors^14^. TSPs also reduce functional postsynaptic AMPA-glutamate receptor accumulation^15^. It was found that postsynaptic expression of α_2_δ-1 is required for TSP-induced synaptogenesis in the CNS, and this was reported to be independent of the function of α_2_δ-1 as a calcium channel subunit^16^. Furthermore, TSPs 1, 2 and 4 were demonstrated to interact with α_2_δ-1 by co-immunoprecipitation from cerebral cortex^16^. The epidermal growth factor (EGF)-like repeats of TSPs were identified to represent their synaptogenic domain, and a synaptogenic region of TSP2 containing these EGF repeats was found to interact with full length α_2_δ-1 and with its VWA domain, when both were co-expressed in HEK-293 cells^16^. In addition, the α_2_δ-1 ligand, gabapentin, was observed to inhibit co-immunoprecipitation between the synaptogenic domain of TSP2 and α_2_δ-1, when they were co-expressed^16^.

In the present study, our aim was to examine whether the gabapentin-sensitive interaction between TSPs and α_2_δ-1 demonstrated previously^16^ could reciprocally affect ^3^H-gabapentin binding. We primarily concentrated on TSP4, as, like α_2_δ-1^8^,^17^, it is up-regulated in dorsal spinal cord following peripheral sensory nerve injury^18^. We therefore performed radioligand binding experiments to examine whether TSP4 affected ^3^H-gabapentin binding to α_2_δ-1, which might influence the efficacy of this drug. We also performed co-immunoprecipitation and immunocytochemical experiments to examine whether α_2_δ-1 and TSP4 interacted with each other in this system.

Our ligand binding experiments show that co-expression of full length TSP4 modestly reduced the binding affinity for ^3^H-gabapentin, and only in the presence of Mg^2+^, whereas the isolated TSP4 EGF domains did not. Furthermore although we were able to demonstrate partial co-immunoprecipitation of α_2_δ-1 and full length TSP4, this did not occur for the EGF domains of TSP4. In immunocytochemistry experiments we could not demonstrate co-localisation of α_2_δ-1 and TSP4 at the cell surface of transfected cells, although both α_2_δ-1 and TSP4 could be detected intracellularly and TSP4 was secreted from transfected cells, in the proximity of cells expressing α_2_δ-1 on the cell surface.

## Results

### Effect of TSP4 on ^3^H-gabapentin binding to α2δ-1

**Figure 1:**
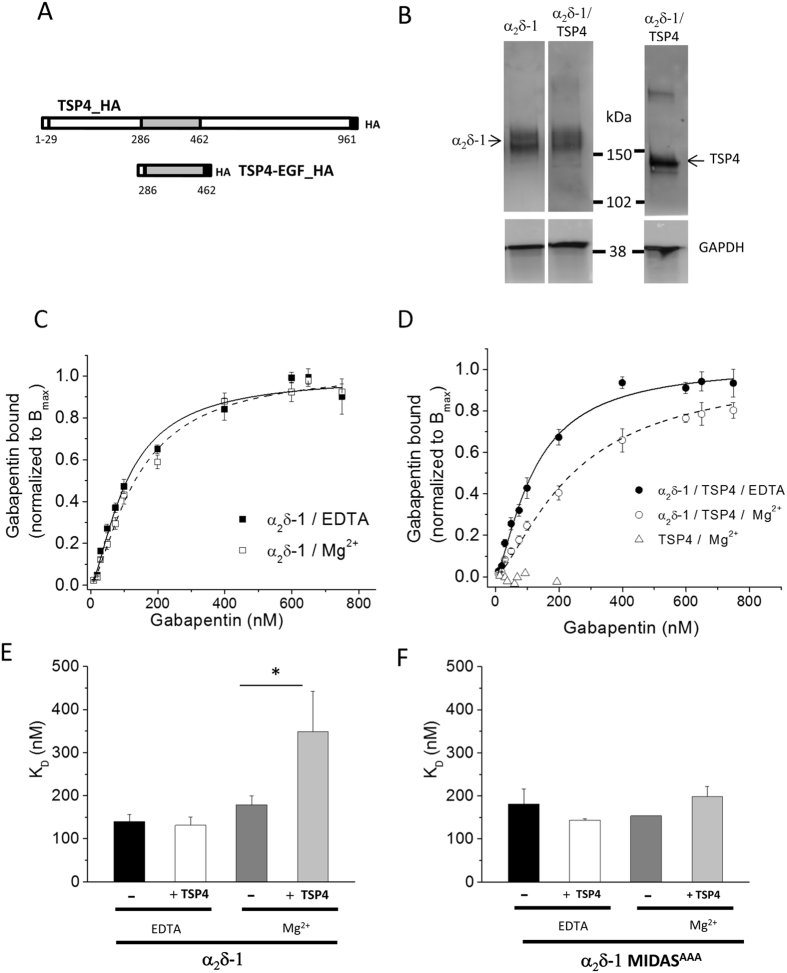
Co-expression of TSP4 and α_2_δ-1 reduces binding affinity of ^3^H-gabapentin to α_2_δ-1 in the presence of Mg^2+^. (**A**) Diagram of TSP constructs used in this study. (**B**) Immunoblot showing expression of α_2_δ-1 alone (left panel) and with TSP4 (middle panel). Corresponding immunoblot for TSP4: right panel (HA Ab). Lower panels: GAPDH loading controls. Protein loaded 15 μg. (**C**) Mean data for [^3^H]-gabapentin binding to α_2_δ-1 without TSP4 in presence of 2 mM Mg^2+^ (open symbols) or EDTA (solid symbols). Mean data from 5 assays (in triplicate) fitted with Hill equation. Data normalized to each mean B_max_ to illustrate difference in K_D_ values. With Mg^2+^, K_D_ = 138.5 nM; *n*_*H*_ = 1.45; with EDTA, K_D_ = 107.8 nM; *n*_*H*_ = 1.48. (**D**) Mean data for [^3^H]-gabapentin binding to α_2_δ-1 with TSP4 in presence of 2 mM Mg^2+^ (open symbols) or EDTA (solid symbols). Mean data from 5 complete sets of experiments (in triplicate) fitted with Hill equation. No binding of ^3^H-gabapentin to TSP4 alone (1 experiment in triplicate, open triangles, normalized to B_max_ of α_2_δ-1). Data normalized to each mean B_max_ to illustrate difference in K_D_. In the presence of Mg^2+^, K_D_ = 243.3 nM; *n*_*H*_ = 1.42; in presence of EDTA, K_D_ = 120.7 nM; *n*_*H*_ = 1.41. (**E**) Mean K_D_ (±SEM) from all experiments with WT α_2_δ-1 and TSP4. α_2_δ-1/EDTA (black, n = 7) α_2_δ-1/TSP4/EDTA (white, n = 7), α_2_δ-1/Mg^2+^ (dark-grey, n = 6) and α_2_δ-1/TSP4/Mg^2 +^ (light-grey, n = 7). The mean B_max_ values (pmol/mg protein) were 2.75 ± 0.69 for α_2_δ-1 with EDTA; 2.39 ± 0.61 for α_2_δ-1 + TSP4 with EDTA; 0.94 ± 0.15 for α_2_δ-1 with Mg^2+^ and 1.09 ± 0.22 for α_2_δ-1 + TSP4 with Mg^2+^. Respective Hill coefficients (*n*_*H*_) were 1.27 ± 0.05, 1.39 ± 0.06, 1.3 ± 0.07 and 1.29 ± 0.09. Statistical analysis performed between paired α_2_δ-1/Mg^2+^ and α_2_δ-1/TSP4/Mg^2+^ data, paired t test, *P = 0.018. (**F**) Mean values for K_D_ (±SEM) for three datasets with α_2_δ-1-MIDAS^AAA^ and TSP4. α_2_δ-1-MIDAS^AAA^/EDTA (black, n = 3); α_2_δ-1-MIDAS^AAA^/TSP4/EDTA (white, n = 3); α_2_δ-1-MIDAS^AAA^ /Mg^2+^ (dark-grey, n = 2; individual values 116.6; 190.4) and α_2_δ-1-MIDAS^AAA^/TSP4/Mg^2+^ (light-grey bar, n = 3). Mean B_max_ (pmol/mg protein) were 0.68 ± 0.12 for α_2_δ-1-MIDAS^AAA^ with EDTA; 0.24 ± 0.02 for α_2_δ-1-MIDAS^AAA^ + TSP4 with EDTA, 0.46 (0.53, 0.38) for α_2_δ-1-MIDAS^AAA^ with Mg^2+^ and 0.37 ± 0.06 for α_2_δ-1-MIDAS^AAA^ + TSP4 with Mg^2+^. Respective *n*_*H*_ were 1.33 ± 0.07, 1.49 ± 0.10, 1.43 (1.55, 1.3) and 1.45 ± 0.05.

It was demonstrated previously that antibodies to TSP1, 2 and 4 can immunoprecipitate α_2_δ-1 from brain^16^. In that study α_2_δ-1 could also be co-immunoprecipitated with a TSP2 synaptogenic region containing its EGF domains, when they were both co-expressed in HEK-293 cells. It was then shown that this co-immunoprecipitation was reduced in the presence of the α_2_δ ligand gabapentin^16^. In order to examine whether such an interaction could reciprocally affect ^3^H-gabapentin binding to α_2_δ-1, we examined the effect of TSP4 because, like α_2_δ-1, it is implicated in neuropathic pain models^18^. We therefore expressed α_2_δ-1 and TSP4 together in tsA-201 cells and performed ^3^H-gabapentin binding assays on membrane preparations. The two main constructs used in these experiments were TSP4_HA (termed TSP4) or its truncated form containing the EGF domains (TSP4-EGF_HA, termed TSP4-EGF) (Fig. 1A). In all preparations used for binding assays, the amount of α_2_δ-1 expressed was also examined by western blotting; α_2_δ-1 was expressed to a similar extent in the absence and presence of TSP4 (Fig. 1B). Since TSPs were reported to interact with the VWA domain of α_2_δ-1^16^, and VWA domains bind protein ligands in a divalent cation-dependent manner^19^,^20^, we performed ^3^H-gabapentin binding in the absence of divalent cations (2 mM EDTA to chelate all divalent cations), or in the presence of 2 mM Mg^2+^ (Fig. 1C,D). We found that the affinity of ^3^H-gabapentin binding to α_2_δ-1 was significantly reduced by the presence of TSP4, but only in the presence of Mg^2+^, and not in the presence of EDTA (Fig. 1C,D). This is shown by the increased K_D_ value in Mg^2+^ (Fig. 1E). In control experiments we observed no direct binding of ^3^H-gabapentin to membranes of tsA-201 cells expressing only TSP4 (Fig. 1D).

We then utilised an α_2_δ-1 construct containing three point mutations in the metal ion dependent adhesion site (MIDAS) motif of the VWA domain^11^. Mutation of the MIDAS motif has been reported to prevent divalent cation-mediated interaction of VWA domains with their protein ligands^21^. We found that α_2_δ-1 MIDAS^AAA^ bound ^3^H-gabapentin with an affinity similar to WT α_2_δ-1 (Fig. 1F). However, no effect of TSP4 was observed on the affinity of ^3^H-gabapentin binding to α_2_δ-1 MIDAS^AAA^, determined in the presence or absence of Mg^2+^ (Fig. 1F). This supports the possibility that TSP4 can interact with the VWA domain of α_2_δ-1 in a Mg^2+^ -dependent manner, and that this interaction allosterically reduces the affinity of ^3^H-gabapentin binding to α_2_δ-1, but does not affect the B_max_.

**Figure 2:**
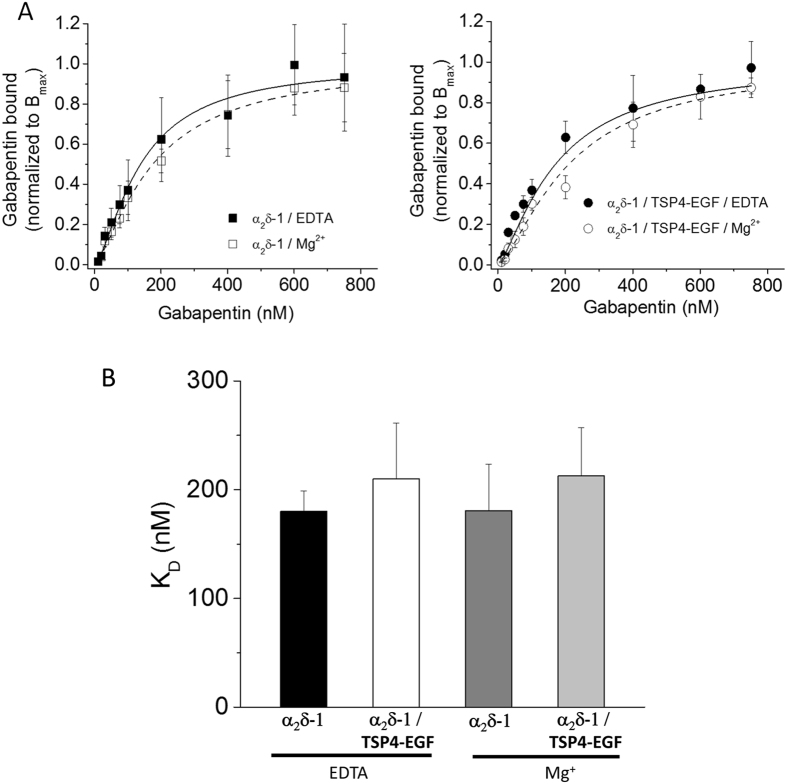
Co-expression of TSP4-EGF domain has no effect on the binding affinity of ^3^H-gabapentin to α_2_δ-1. (**A**) Left: Saturation binding isotherms show the mean data obtained for [^3^H]-gabapentin binding toα_2_δ-1 in the presence of 2 mM Mg^2+^ (open squares) or EDTA (solid squares). Mean data from 3 independent experiments (each in triplicate) were fitted using the Hill equation, and normalized to the mean B_max_ values (dashed and solid lines respectively). In the presence of Mg^2+^, K_D_ for the mean data = 175.2 nM and *n*_*H*_ = 1.39; in the presence of EDTA, K_D_ for the mean data = 138.5 nM and *n*_*H*_ = 1.48. The mean B_max_ values (pmol/mg protein) were 0.71 ± 0.04 for α_2_δ-1 with Mg^2+^ and 0.90 ± 0.4 for α_2_δ-1 with EDTA. Right: Saturation binding isotherms show the mean data obtained for [^3^H]-gabapentin binding to α_2_δ-1 in the presence of TSP4-EGF in the presence of 2 mM Mg^2+^ (open circles) or EDTA (solid circles). Mean data from the same 3 independent experiments (each in triplicate) as above were fitted using the Hill equation, and normalized to the mean B_max_ values (dashed and solid lines respectively). In the presence of Mg^2+^, K_D_ for the mean data = 213.0 nM and *n*_*H*_ = 1.44; in the presence of EDTA, K_D_ = 172.6 nM and *n*_*H*_ = 1.37. The mean B_max_ values (pmol/mg protein) were 0.55 ± 0.05 for α_2_δ-1 + TSP4-EGF with Mg^2+^ and 0.79 ± 0.14 for α_2_δ-1 + TSP4-EGF with EDTA. In both the presence and absence of TSP4-EGF, the B_max_ for ^3^H-gabapentin binding was reduced by Mg^2+^. (**B**) The mean values (±SEM) for K_D_ calculated from fitting the individual experiments contributing to data in (**A,B**) (n = 3). α_2_δ-1/EDTA (black bar) α_2_δ-1/TSP4-EGF/EDTA (white bar), α_2_δ-1/Mg^2+^ (dark grey bar) α_2_δ-1/TSP4-EGF/Mg^2+^ (light grey bar). Statistical analysis performed using one-way ANOVA, showed no statistical differences.

The EGF-like domains of TSPs were identified previously as their synaptogenic region, and for TSP2 a domain including the EGF repeats was found to be the region interacting with α_2_δ-1^16^. We therefore examined the ability of an equivalent TSP4-EGF domain construct to affect ^3^H-gabapentin binding to α_2_δ-1. However, we found no effect of this domain on the affinity of ^3^H-gabapentin binding to α_2_δ-1, either in the presence or absence of Mg^2+^ (Fig. 2A,B). In a control experiment, TSP4-EGF alone showed no ^3^H-gabapentin binding (data not shown).

### Examination of the presence of TSP4 with α_2_δ-1 in DRM fractions

**Figure 3:**
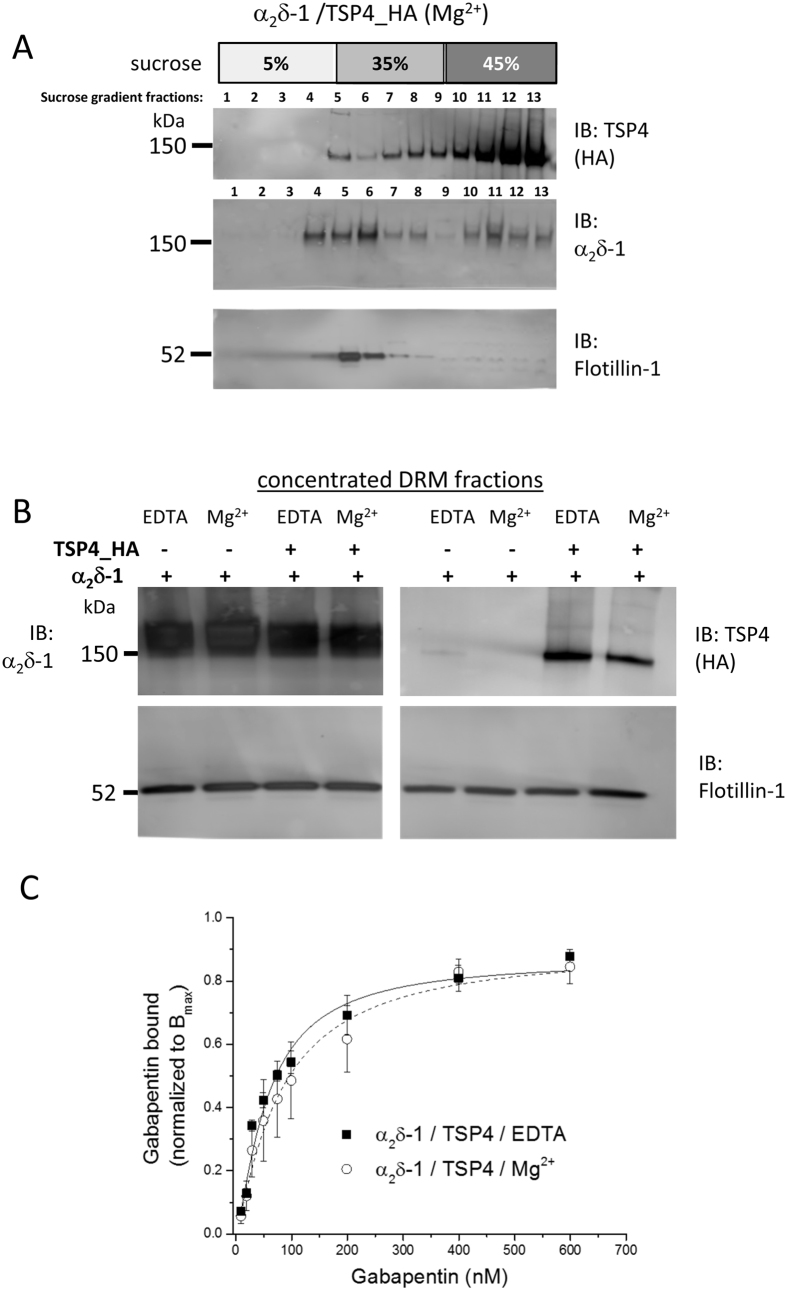
Distribution of TSP4 and α_2_δ-1 in DRMs, and effect of TSP4 on affinity of binding of ^3^H-gabapentin to α_2_δ-1 in DRMs. (**A**) Representative Western blots of DRMs prepared in the presence of Mg^2+^ showing that TSP4 was mainly concentrated in the detergent-soluble fractions (top panel, lanes 10–13) although a small proportion of TSP4 is present in DRM fraction 5, whereas α_2_δ-1 is concentrated in DRM fractions (middle panel). The bottom blot shows the location of the DRM fraction marker flotillin-1 (lanes 4–6). 5 μl aliquots loaded/lane. (**B**) Aliquots (4 μg protein) from the DRM fractions shown in (**A**). The presence of Mg^2+^ during the preparation of the sucrose gradient separation did not affect the distribution of α_2_δ-1 (upper left panel) and TSP4 (upper right panel) in the DRM fractions. The lower panel of each western blot shows the DRM marker flotillin-1. (**C**) Saturation binding isotherms show the mean data obtained for [^3^H]-gabapentin binding to DRMs from cells expressing α_2_δ-1 and TSP4 with EDTA (n = 3, solid squares) or Mg^2+^ (n = 4, open circles). Mean normalised data from independent experiments (each in triplicate) were fitted using the Hill equation. The mean K_D_ values from fitting the individual experiments were 85.8 ± 12.8 nM for α_2_δ-1/TSP4/EDTA and 125.9 ± 40.3 nM for α_2_δ-1/TSP4/Mg^2+^. The respective B_max_ (pmol/mg protein) were 43.6 ± 13.0 and 13.8 ± 4.6, and the respective *n*_*H*_ values were 1.00 ± 0.10 and 1.24 ± 0.18.

The α_2_δ proteins are strongly concentrated in detergent-resistant membrane (DRM) fractions^22^,^23^,^24^ (Fig. 3A). For this reason we examined whether TSP4 would co-purify with α_2_δ-1 in DRMs, as evidence of an interaction. When co-expressed with α_2_δ-1 in tsA-201 cells, only a very small proportion (about 7% from the experiment shown in Fig. 3A) of TSP4 was also observed in DRM fraction 5 (Fig. 3A). This distribution was not affected by DRM preparation in the presence or absence of Mg^2+^, as shown by Western blotting of α_2_δ-1 and TSP4 from concentrated DRMs (Fig. 3B). To examine whether the amount of TSP4 that co-localises with α_2_δ-1 in DRMs is sufficient to affect the binding of gabapentin to α_2_δ-1, ^3^H-gabapentin binding was determined in these DRMs. The observed K_D_ for ^3^H-gabapentin binding in DRMs in Mg^2+^ -containing medium was unchanged by the presence of TSP4 (Fig. 3C), and was similar to that found previously in DRMs^25^. This result indicates that the amount of TSP4 that is present with α_2_δ-1 in the DRMs does not cause a reduction in the binding affinity of gabapentin to α_2_δ-1.

We then investigated whether we could demonstrate any evidence for an effect of TSP4 on ^3^H-gabapentin binding in brain. The binding affinity of ^3^H-gabapentin is known to increase successively as α_2_δ-1 is purified from brain membranes^26^. We wished to test the hypothesis that an interaction between TSPs and α_2_δ-1 might contribute to the lower ^3^H-gabapentin binding affinity in brain membranes compared to the purified α_2_δ-1 protein. To do this, we took advantage of our finding that the reduction of ^3^H-gabapentin binding affinity in the presence of TSP4 that we observed in tsA-201 cell membranes was dependent on the presence of Mg^2+^, and was not observed in the presence of EDTA.

We therefore examined whether there was a Mg^2+^ -dependent effect on ^3^H-gabapentin binding affinity in brain, which would be compatible with a Mg^2+^ -dependent interaction between an endogenous binding partner (such as TSP4) and endogenous α_2_δ-1. However, in an experiment using adult rat brain tissue, we found that the ^3^H-gabapentin binding affinity was not altered by the presence of EDTA or MgCl_2_, either in crude brain membranes (K_D_ 217.9 nM and 189.1 nM, respectively; Supplementary Fig. S1A) or in DRMs prepared from brain (K_D_ 40.9 nM and 52.7 nM, respectively; Supplementary Fig. S1B). As previously observed in tsA-201 cells, the presence of Mg^2+^ reduced the B_max_ for ^3^H-gabapentin in brain membranes. This suggests that the ^3^H-gabapentin binding affinity of α_2_δ-1 in brain membranes is not influenced by the interaction of any proteins (including TSPs) binding to the VWA domain of α_2_δ-1 in a Mg^2+^ -dependent manner.

### Interaction between α2δ-1 and TSP4

**Figure 4:**
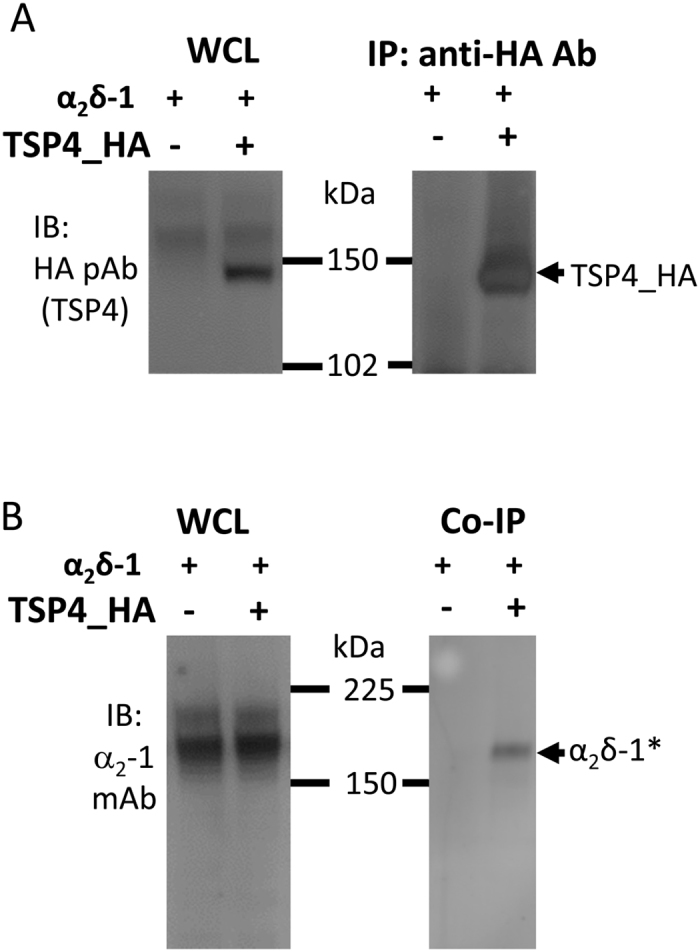
Western blot analysis of co-immunoprecipitation of α_2_δ-1 with TSP4. (**A**) Immunoblots of WCL (15 μg protein, left) and immunoprecipitation (15 μl aliquot, containing 0.5 μg protein, right) of TSP4_HA with rabbit anti HA antibody from cells transfected with α_2_δ-1 and TSP4_HA (right lane), but not when transfected with α_2_δ-1 alone (left lane). (**B**) Immunoblots of WCL (15 μg protein, left) and co-immunoprecipitation (co-ip, 15 μl, right) of α_2_δ-1 (*) with rabbit anti HA antibody from cells co-transfected with TSP4_HA (right lane), but not when transfected with α_2_δ-1 alone (left lane).

In order to examine whether there was an interaction between α_2_δ-1 and TSP4 in our system, and to understand where in the cell this putative interaction was occurring we also performed co-immunoprecipitation and immunocytochemistry. For co-immunoprecipitation studies, we expressed α_2_δ-1 and TSP4_HA together in tsA-201 cells. Full-length TSP4 was well-expressed (Fig. 4A) and could be immunoprecipitated with HA antibody (Fig. 4B). We observed a small amount of co-immunoprecipitation of α_2_δ-1 with full-length TSP4, but the proportion of α_2_δ-1 that was co-immunoprecipitated with TSP4 was very low, compared to that in the WCL (Fig. 4B), and compared to the amount of TSP4 which was efficiently immunoprecipitated. Co-immunoprecipitation of α_2_δ-1 with TSP4 was observed in 8/13 experiments; the amount of co-immunoprecipitation of α_2_δ-1 in these experiments is quantified relative to TSP4 immunoprecipitated in Supplementary Table S1. These results indicate that the interaction between α_2_δ-1 and full length TSP4 when they are co-expressed is probably weak, in agreement with the small amount of TSP4 co-purifying with α_2_δ-1 in DRM fractions.

No specific co-immunoprecipitation of α_2_δ-1-MIDAS^AAA^ was seen with TSP4 (Supplementary Fig. S2A–D, lane 2). Furthermore, we did not observe co-immunoprecipitation of α_2_δ-1 with TSP4-EGF_HA (Supplementary Fig. S2, lane 4). These results agree with the lack of effect of TSP4 on ^3^H-gabapentin binding to α_2_δ-1-MIDAS^AAA^ (Fig. 1F), and the lack of effect of TSP4-EGF domain on ^3^H-gabapentin binding to α_2_δ-1 (Fig. 2A,B).

**Figure 5:**
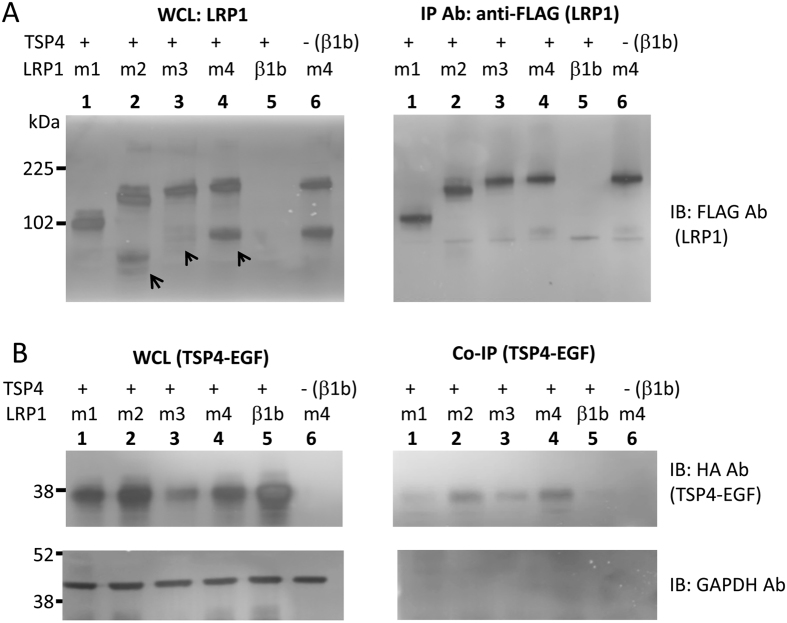
The EGF domain of TSP4 binds to LRP1. (**A**,**B**) Western blot analysis of the immunoprecipitation of LRP1_FLAG constructs (**A**) and co-immunoprecipitation of TSP4-EGF_HA (**B**), for LRPm1_FLAG (lane 1), LRPm2_FLAG (lane 2), LRPm3_FLAG (lane 3) or LRPm4_FLAG (lane 4) and, as negative controls, the auxiliary calcium channel subunit β1b and TSP4-EGF_HA (lane 5) or LRPm4_FLAG and β1b (lane 6). The WCL (15 μg protein for both panels, left) and immunoprecipitated fractions (15 μl aliquots, for both panels, right) were analysed by Western blot with monoclonal anti-FLAG antibody (**A**) and anti-HA antibody (**B**, upper panels). GADPH was used as loading control, and control for immunoprecipitation ((**B**), lower panels). The arrowheads in the upper panels indicate the cleaved forms of the LRP1 domains, which correspond to the N-terminal ligand-binding domain subunit. The LRP1 immunoreactive bands at higher molecular weight correspond to the uncleaved form of LRPm1-4 (~120 kDa to ~200 kDa). The MW markers refer to both panels. The results shown are representative of 4 independent experiments.

TSPs have been documented to bind to a number of proteins including the cell surface receptor LRP1^27^,^28^. LRP1 contains four ligand binding domains^29^, which can each be separately expressed as a minigene fused with the LRP1 transmembrane domain, and retain ligand binding activity^30^,^31^. As a control, we immunoprecipitated these constructs using the same immunoprecipitation protocol, and found that TSP4-EGF (Fig. 5) and full length TSP4 (Supplementary Fig. S3) co-immunoprecipitated robustly with LRP1 ligand binding domains.

### Immunocytochemical investigation of distribution of expressed α_2_δ-1 with TSP4 in tsA-201 cells

**Figure 6:**
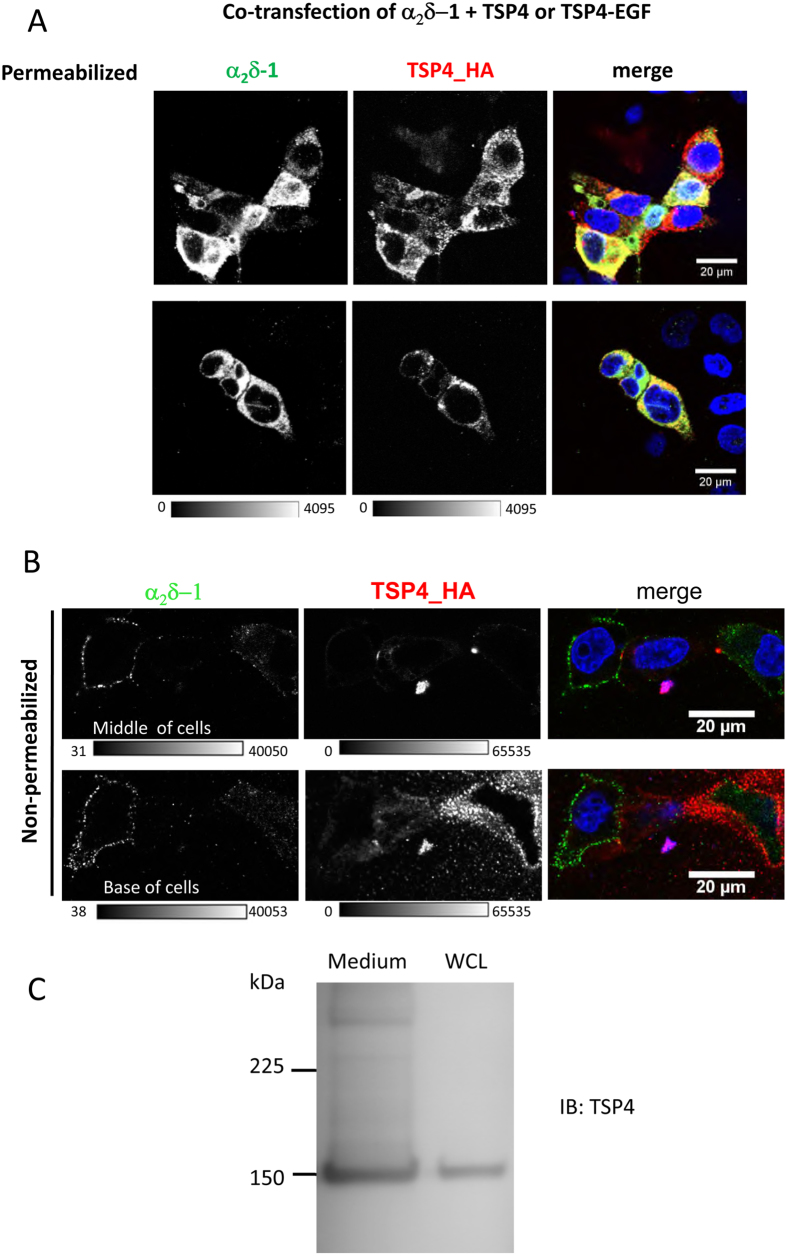
Immunocytochemical detection of co-expressed TSP4 and α_2_δ-1. (**A**) Representative images of permeabilised tsA-201 cells co-expressing α_2_δ-1 (left, grey-scale) and TSP4_HA constructs (middle, grey-scale). Upper panel: full length TSP4_HA, lower panel TSP4-EGF_HA. The grey-scale calibration applies to both panels. Right panel shows merged image, with yellow showing the presence of both red and green in individual pixels. DAPI was used to visualise the nucleus (blue). (**B**) Representative images of non-permeabilised tsA-201 cells co-expressing α_2_δ-1 (left, grey-scale) and TSP4_HA (middle, grey-scale). The upper panel is an image through the center of the cells, and the lower panel is at the base of the cells in the same field of view. Right panels show merged images (α_2_δ-1, green; and TSP4-HA, red); yellow would show the presence of both red and green in individual pixels. DAPI was used to visualise the nucleus (blue). The grey-scale calibration bar is shown below each image. For both (**A**,**B**), scale bars are 20 μm. Images are from 3 independent experiments. (**C**) Immunoreactive band for TSP4 (using TSP4 Ab) at ~150 kDa was detected in 5 μl concentrated culture medium (left lane) from tsA-201 cells transfected with TSP4 (WCL in right lane).

TSPs are secreted extracellular matrix proteins. In order to determine whether secreted TSP4 was able to bind to α_2_δ proteins expressed on the cell surface of transfected cells, mimicking what might occur in native tissues, we first co-transfected α_2_δ-1 and TSP4 into tsA-201 cells. TSP4 protein was detected by immunoreactivity to its HA tag. As expected, the two proteins were both found to be present intracellularly in permeabilised cells when they were co-transfected (Fig. 6A, upper row). Similarly, TSP4-EGF and α_2_δ-1 could both be detected intracellularly when co-transfected (Fig. 6A, lower row).

In non-permeabilised cells, we observed secreted TSP4 spreading around the base of co-transfected cells, associated with the substrate on the coverslip, whereas α_2_δ-1 was expressed on the cell surface (Fig. 6B, lower row). In contrast, at a higher focal plane, there was very little TSP4 in the proximity of the cell surface, where α_2_δ-1 was observed (Fig. 6B, upper row), although in the same experiment, but in permeabilising conditions, both were again detected intracellularly (data not shown). TSP4 was also detected in the medium (Fig. 6C), further evidence that it was secreted from transfected tsA-201 cells. In none of these experiments did we observe TSP4 co-localised with α_2_δ-1 on the cell surface. Furthermore, fewer cells were observed expressing α_2_δ-1 on the cell surface when TSP4 was co-expressed, suggesting α_2_δ-1 may have been retained intracellularly by co-expression with TSP4.

**Figure 7:**
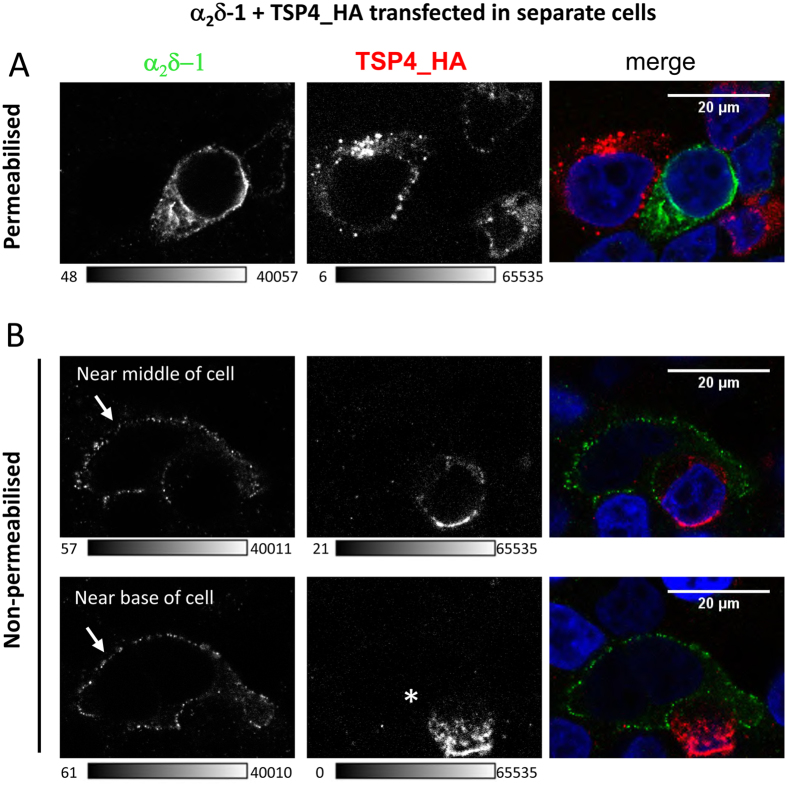
Immunocytochemical detection of TSP4 and α_2_δ-1 expressed in separate populations of cells. (**A**,**B**) Representative images of neighbouring tsA-201 cells transfected separately and expressing α_2_δ-1 (left, grey scale) or TSP4-HA (middle, grey-scale). Right panel shows merged image (α_2_δ-1, green; TSP4-HA, red). DAPI was used to visualise the nucleus (blue). TSP4-expressing cells were layered onto α_2_δ-1-expressing cells that had already adhered to poly-lysine coated coverslips. Cells were either permeabilised (**A**) or non-permeabilised (**B**), upper row shows an optical section near the center of the cell and lower row shows a plane near the bottom of the same cell. Arrow indicates α_2_δ-1 on cell surface). Scale bars are 20 μm. Images are representative of cells from 3 independent experiments. *Indicates secreted TSP4, near to cells expressing α_2_δ -1. The grey-scale calibration bar is shown below each image.

In order to mimic the situation in the brain where TSP4 is secreted mainly from astrocytes and α_2_δ-1 is on the cell surface of neurons, in the next set of experiments we transfected α_2_δ-1 and TSP4 separately, and then mixed the two populations of transfected cells (Fig. 7). However, again no co-localisation was observed on the cell surface of closely-apposed cells. When we permeabilised cells, and examined the region where two neighboring cells bordered each other, one expressing α_2_δ-1 and the other secreting TSP4 (Fig. 7A), there was no co-localisation on the bordering cell surface (right panel). Furthermore in non-permeabilised cells (Fig. 7B), no co-localisation of α_2_δ-1 with TSP4 was seen around α_2_δ-1-expressing cells (arrow indicates α_2_δ-1 on cell surface), despite the clear presence of a neighbouring cell secreting TSP4 (*Fig. 7B, the lower middle panel shows the base of the cells with secreted TSP4).

**Figure 8:**
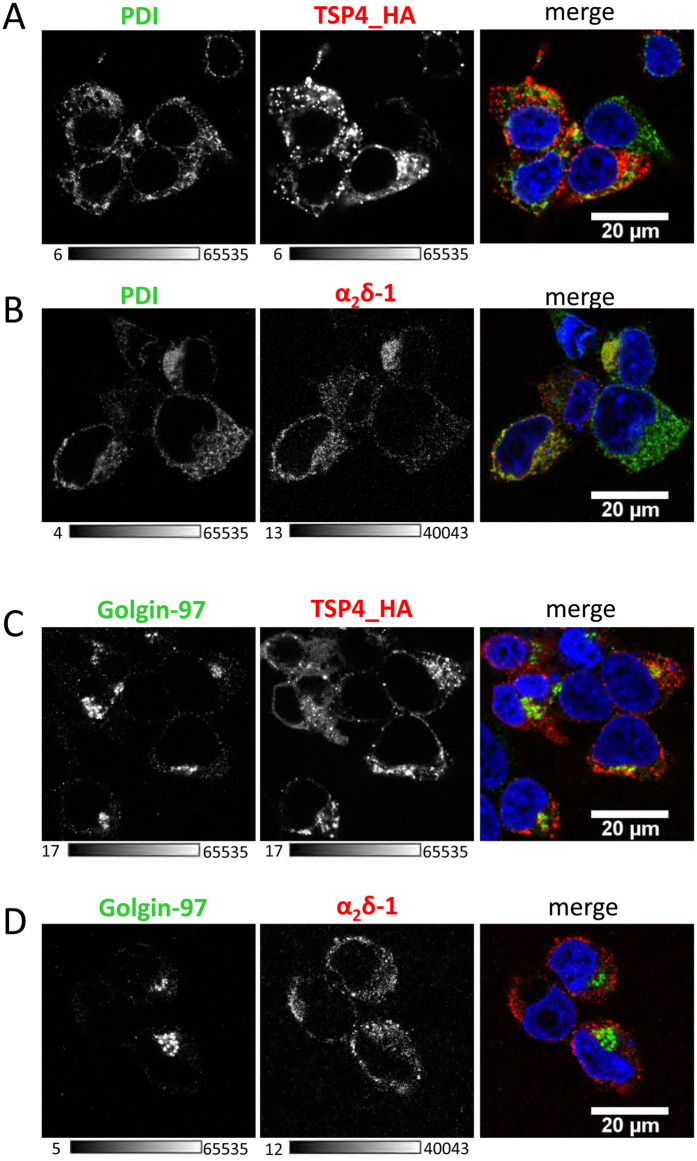
Immunocytochemical detection of co-expressed TSP4 or α_2_δ-1 with subcellular markers for ER and Golgi (PDI and Golgin-97). Representative images of permeabilised tsA-201 cells co-expressing TSP4-HA and α_2_δ-1 and then probed with the following antibodies: (**A**) PDI (left, grey-scale) and TSP4_HA (middle, grey-scale), (**B**) PDI (left, grey-scale) and α_2_δ-1 (middle, grey-scale), (**C**) Golgin-97 (left, grey-scale) and TSP4_HA (middle, grey-scale), (**D**) Golgin-97 (left, grey-scale) and α_2_δ-1 (middle, grey-scale). Right panels show merged images (PDI or Golgin-97, green; α_2_δ-1 or TSP4-HA, red); with yellow showing the presence of both red and green in individual pixels. DAPI was used to visualise the nucleus (blue). Scale bars 20 μm. The rabbit α_2_δ-1 polyclonal antibody was used in this experiment. Images are representative of >100 cells for each condition. The grey-scale calibration bar is shown below each image.

In order to determine the intracellular compartments in which α_2_δ-1 and TSP4 were localised we performed immunocytochemistry experiments using antibodies against markers of the endoplasmic reticulum (ER) and Golgi apparatus. In permeabilised cells, when both TSP4 and α_2_δ-1 were transfected together, TSP4 was co-localised in part with both the ER marker PDI and the Golgi marker Golgin-97, but also showed extensive punctate localisation near the plasma membrane, which was not associated with ER or Golgi (Fig. 8A,C). In contrast, α_2_δ-1 was extensively co-localised with the ER marker but very little was associated with the Golgi (Fig. 8B,D).

## Discussion

The α_2_δ proteins were first identified as auxiliary subunits of voltage gated calcium channels, affecting both their trafficking and biophysical properties (for review see^1^), but more recently they have also been proposed to play other roles, independent of their involvement in calcium channel function^16^,^32^,^33^. Nevertheless, since α_2_δ proteins traffic calcium channels to the plasma membrane and to presynaptic terminals as well as affecting channel function^9^,^11^, it is difficult to separate their independent roles from their roles as calcium channel auxiliary subunits.

In view of the fact that the gabapentinoid drugs, gabapentin and pregabalin, bind to α_2_δ-1 and α_2_δ-2 proteins^1^,^3^,^4^,^5^, the potential of these proteins to have multiple functions is of translational importance. The affinity of gabapentin binding has been shown to depend on the state of purification of α_2_δ-1, being reported to be 92 nM in brain membranes and 9.4 nM in purified protein^26^, reflecting the possibility that an endogenous small molecule binding partner is lost during purification. In agreement with this we have found here that the binding affinity for ^3^H-gabapentin is increased in purified DRM fractions from brain.

Gabapentin interacts with a binding site found in both α_2_δ-1 and α_2_δ-2, involving three arginine residues, upstream of the VWA domain^3^,^24^,^34^. In agreement with this, we have found here that gabapentin still binds to α_2_δ-1 MIDAS^AAA^ with a similar affinity to that observed for WT α_2_δ-1, indicating that VWA domain function is not directly involved in the binding of gabapentin. We also demonstrated a similar result for α_2_δ-2^10^. It was previously found that gabapentin was able to prevent co-immunoprecipitation between α_2_δ-1 and a TSP2 synaptogenic domain when they were co-expressed, and that the interaction involved the α_2_δ-1 VWA domain^16^. Therefore gabapentin could be considered to be an allosteric modulator of TSP binding to α_2_δ-1, since gabapentin itself does not bind directly to the VWA domain. Allosteric interactions between two sites are normally reciprocal^35^, and therefore we hypothesized that the interaction of TSPs with α_2_δ-1 might influence ^3^H-gabapentin binding. Indeed, we found this to be the case; in membrane preparations prepared from co-transfected cells, the presence of TSP4 significantly reduced the affinity for ^3^H-gabapentin binding to α_2_δ-1, although this result was not observed in more purified DRM fractions, where little TSP4 co-purified with α_2_δ-1. The effect of TSP4 on ^3^H-gabapentin binding was only observed in the presence of Mg^2+^, not when divalent cations were chelated. Furthermore, it was absent when using α_2_δ-1 MIDAS^AAA^, indicating that it is likely to be mediated by the interaction of TSP4 with the α_2_δ-1 MIDAS motif, in a Mg^2+^ -dependent manner.

In summary, our studies indicate that if α_2_δ-1 and TSP4 are both expressed in the same cells and are therefore present at high concentration intracellularly, then TSP4 can influence the properties of α_2_δ-1, in terms of ^3^H-gabapentin binding affinity. However, this effect was not reproduced by the EGF repeat domain of TSP4, which is comparable to the region of TSP2 previously found to interact with α_2_δ-1^16^, although the TSP4-EGF construct has four, rather than three EGF repeats, and it does not contain a properdin-like domain.

In the co-immunoprecipitation experiments presented here, using C-terminally HA-tagged TSP4, some co-immunoprecipitation with co-expressed full length α_2_δ-1 was found, although this was not observed in all experiments and was not reproduced with the TSP4-EGF domain. This suggests that the interaction between α_2_δ-1 and TSP4 is probably of low affinity, and this is in agreement with our finding that the effect of TSP4 on ^3^H-gabapentin binding is absent in DRM preparations, where any interaction between α_2_δ-1 and TSP4 is not retained, as there is very little co-purification between α_2_δ-1 and TSP4. In agreement with this, the affinity of ^3^H-gabapentin binding to brain membranes and brain DRMs was not affected by chelating divalent cations with EDTA, which would disrupt any interaction between endogenous α_2_δ-1 and TSPs, indicating that TSPs do not represent a significant native interaction partner of α_2_δ-1 in adult rat brain, despite the presence of TSP4 in this tissue^36^.

Co-immunoprecipitation was previously demonstrated of the TSP2 synaptogenic domain with α_2_δ-1 or its VWA domain when the two proteins were co-expressed in HEK-293 cells, using C-terminal FLAG or other tags on α_2_δ-1 for the immunoprecipitation^16^. We have previously observed that C-terminal tags were cleaved from α_2_δ subunits during their processing, and were not associated with cell surface α_2_δ subunits^23^. This was one of the initial supporting pieces of evidence for glycosylphosphatidylinositol (GPI)-anchoring of the α_2_δ proteins^23^. Furthermore, the vast majority of transfected α_2_δ-1 expressed in tsA-201 cells is not on the cell surface, as also demonstrated in cell surface biotinylation experiments^22^. Therefore immunoprecipitation of the C-terminal FLAG-tagged α_2_δ-1 with co-expressed TSPs^16^ is likely to be occurring mainly between intracellular proteins. This is also true for the co-immunoprecipitation studies performed here, since in these experiments the two proteins were co-expressed, and we have shown that both are in part associated with the ER compartment, as expected. Furthermore, we have obtained no evidence from the immunocytochemical experiments presented here that TSP4, once secreted, interacts with α_2_δ-1 on the cell surface of the same or adjacent cells. It is of course possible that additional factors, not present in the transfected cells, are required for such an interaction. Of interest, it has recently been identified that TSPs interact intracellularly with the ER-resident protein stromal interaction molecule-1^37^.

α_2_δ-1 is upregulated in DRG neurons following peripheral sensory nerve injury^8^,^38^,^39^, and there is increased trafficking of α_2_δ-1 to presynaptic terminals in the dorsal horn of the spinal cord^8^. TSPs are mainly expressed and secreted by astrocytes in the CNS, including the spinal cord^14^,^18^, and TSP4 is secreted by astrocytes in the subventricular zone after brain injury^40^. TSP4 is also up-regulated in dorsal spinal cord following peripheral sensory nerve injury in rats, and this up-regulation correlates with the development of neuropathic pain^18^. From the ^3^H-gabapentin binding studies described here, one inference might be that the up-regulation of TSP4 in neuropathic pain conditions could limit the effectiveness of gabapentin, as it reduces the affinity of gabapentin binding to α_2_δ-1. However, as described above, in the spinal cord, α_2_δ-1 is present mainly on the surface of presynaptic terminals^8^, whereas TSP4 is a secreted protein, produced primarily by non-neuronal cells^14^,^18^. Thus physiologically or pathologically the two proteins would be likely to interact and affect gabapentin binding to α_2_δ-1 on the cell surface, primarily of the DRG neurons. However in the present study we have been unable to identify a cell surface interaction between TSP4 and α_2_δ-1, although this might occur under conditions not tested here. Interestingly, it has recently been demonstrated that TSP4 is also expressed within DRG neurons and is upregulated in the ganglia following nerve injury^41^. Since we have shown previously that gabapentin reduces the trafficking of α_2_δ-1 to the cell surface^7^, it is therefore possible that TSP4 will interfere with the ability of gabapentin to bind to α_2_δ-1 and reduce calcium channel trafficking, if they are both present in the same cells.

## Materials and Methods

### Molecular biology

The pcDNA3 TSP4_HA construct was generated by PCR from the human TSP4 cDNA. pcDNA3 TSP4-EGF_HA was then generated (containing only the EGF-like repeats after the signal sequence; residues 286–462) with an HA tag on the C terminus, Fig. 1A. LRP1 minigenes 1–4 were also used^42^, and for immunoprecipitation a triple FLAG tag was inserted in place of the original HA tag between residues 24 and 25 of their coding sequences. The α_2_δ constructs used here have been described previously^9^,^10^.

### Cell culture and Transfection

The tsA-201 cells were cultured as described previously^10^,^22^. Cells were transfected using FuGENE 6 reagent (Roche) for all biochemical experiments, or PolyJet (SignaGen) for imaging experiments in tsA-201 cells, according to the manufacturers’ instructions.

### Membrane and detergent-resistant membrane (DRM) preparation

Cell membrane and DRM fractions were prepared essentially as described previously^22^. Cultured tsA-201 cells were washed twice in ice-cold PBS and then left in ice-cold PBS containing Complete protease inhibitor cocktail (Roche, concentration according to manufacturer’s instructions) for 5 min. Cells were gently washed off the bottom of the flask and pelleted at 2000 × g for 10 min. Pellets were kept on ice if used immediately, or stored at −20 °C until required. For brain membranes, adult male Sprague Dawley rats (200–250 g) were euthanised by CO_2_ inhalation and then decapitated. The whole brain minus cerebellum was homogenised on ice in a buffer containing the following (in mM): 20 HEPES, pH 7.4, 2 EDTA, EDTA-free protease inhibitor cocktail (Roche, concentration according to manufacturer’s instructions).

For membrane preparation, pellets obtained from cell harvesting, resuspended in ice-cold 10 mM Hepes plus protease inhibitors (pH 7.4), or brain homogenate, were mechanically lysed by 10–15 passages through a 23 gauge needle. Cell debris and non-lysed cells were pelleted for 15 min at 1,000 × *g*. The supernatant obtained was transferred to a 30 ml thick-walled ultracentrifuge tube (Beckman-Coulter). Membranes were pelleted in an ultracentrifuge (Beckman-Coulter) for 90 min at 60,000 × *g*, and resuspended as required. Aliquots were used to determine the protein concentration. For detection of secreted TSP4, medium was collected from transfected cells and concentrated 15-fold using 10 kDa cut-off filter (GE Healthcare).

For DRM preparation, the method used was essentially as described previously^22^. Briefly, tsA-201 cell pellets or brain homogenate, were resuspended in Mes-buffered saline (MBS: 25 mM Mes, pH 6.5, 150 mM NaCl and EDTA-free protease inhibitor cocktail, containing 1% (v/v) Triton X-100 (Thermo Scientific)), and incubated on ice for 1 h. An equal volume of 90% (w/v) sucrose in MBS was then added to obtain 45% final concentration and overlaid with 35% (w/v) and 5% (w/v) sucrose in MBS to form a discontinuous gradient. The samples were centrifuged at 14,000 × *g* for 18 h at 4 °C (Beckman SW40 rotor). 1 ml fractions were subsequently harvested from the top to the bottom of the tube. When necessary, protein fractions from the gradient were washed free of sucrose by dilution into 25 volumes of PBS and centrifugation (150,000 × *g*, for 1 h at 4 °C) to pellet the DRM material. The material was either used immediately or stored at −20 °C until required.

### ^3^H-Gabapentin binding assay

^3^H-gabapentin binding was performed essentially as described previously^25^. [^3^H]-gabapentin (ARC, St. Louis, MO), was aliquoted and stored at −80 °C. The specific activity was about 110 Ci/mmol and the concentration 1 mCi/ml. Non-radioactive gabapentin (U.S. Pharmacopea or Pfizer) was stored at 4 °C until use and all the solutions and the reagents were kept on ice during the preparation. Binding of [^3^H]-gabapentin was performed in a final volume of 250 μl at room temperature for 90 min. 50 μg/tube of membranes or 3 μg/tube of DRMs were incubated with various concentrations of [^3^H]-gabapentin in 10 mM HEPES/KOH, pH 7.4. Each experiment was carried out in triplicate, and the mean value used for subsequent analysis. Concentrations of [^3^H]-gabapentin higher than 20 nM were achieved by adding non-radioactive gabapentin and correcting the specific binding by the dilution factor (as described previously^4^).

Non-specific binding was determined in the presence of 100-fold or more excess of non-radioactive gabapentin (100 μM). A maximum level of non-specific binding of 25% of total was accepted. Total counts per minute were determined (in duplicate) to give an estimate of the activity of the [^3^H]-gabapentin. Harvesting of the samples was performed by filtration through GF/B filter papers (Whatman, GE Healthcare) pre-soaked in 0.3% polyethyleneimine solution, in order to reduce non-specific binding to the filter paper. The samples were transferred to the filters by washing the tubes three times with Tris-HCl (100 mM, pH 7.4, Sigma 7–9) using a Brandel Harvester (Brandel). The GF/B filter papers were then cut out and transferred to vials containing 5 ml of scintillant (Liquid scintillant cocktail, Beckman Coulter). Samples were equilibrated for 18 h and then the activity was counted in triplicate in a scintillation counter (Beckman Coulter) for 10 min per sample. Data were fit using the Hill- equation (y = B_max_*x^*n*^/(K_D_^*n*^ + x^*n*^), where B_max_ is the total number of binding sites; *n*, termed *n*_*H*_ in the text, is the Hill coefficient and K_D_ is the binding affinity. Hill coefficients were usually >1 and <1.5, which is very common for ligand binding studies, for reasons discussed previously^43^.

### Primary Antibodies

The following primary antibodies were used: for α_2_δ-1 (unless stated) an anti-α_2_δ-1 (mouse monoclonal, Sigma) was used. Where stated, a custom-made rabbit polyclonal antipeptide antibody directed against residues EPFPSAVTIKSWVDK (equivalent to residues 1–15 of mature rat α_2_δ-1 following cleavage of the signal sequence). Other antibodies used were: anti-HA (rabbit polyclonal, Santa Cruz or rat monoclonal, Roche), anti-flotillin-1 (mouse monoclonal, BD Biosciences), anti-GAPDH (mouse monoclonal, Ambion), anti-TSP4 antibody (mouse monoclonal #276523, R&D systems), rabbit polyclonal or mouse monoclonal anti-FLAG antibodies (Sigma), mouse Golgin-97 (Molecular Probes) and mouse Protein disulphide isomerase (PDI, Abcam).

### Co-immunoprecipitation

The tsA-201 cells were transfected with the α_2_δ-1 and TSP4_HA (or non-tagged TSP4 in Fig. 6C) cDNA, or with LRP1 m1-m4_Flag cDNA. Receptor-associated protein (RAP) cDNA was included in all transfections in which LRP1 constructs were used, to ensure their correct folding^44^. Freshly harvested cell pellets were re-suspended in Lysis Buffer (Supplementary Table S2) using first a 200 μl Gilson pipette and then a 23 gauge needle. Samples were then left on ice for 30 min and centrifuged at 14,000 × *g* for 30 min at 4 °C. The supernatant WCL (1 mg protein) was pre-cleared for 2 h at 4 °C with 20 μl of Protein-G agarose beads (Invitrogen) to reduce the non-specific binding to the agarose beads (except in Figs 4,5 and S3, in which Protein-A/G-PLUS agarose (Santa Cruz) were used without a pre-clearing step). The supernatant was recovered by centrifugation at 400 × *g* for 30 s. and then incubated overnight with 5 μg/ml of rabbit anti-HA antibody or mouse anti-FLAG antibody at 4 °C. It was then incubated with Protein-G (or A/G-PLUS) beads for 2 h at 4 °C. A series of washes were carried out in order to reduce the non-specific binding to the beads (Supplementary Table S2). Protein bound to the beads was eluted with loading sample buffer at a final concentration of 1×, containing 100 mM dithiothreitol, followed by incubation for 15 min in a water bath at 55 °C and subsequent centrifugation of the protein-G-beads. Samples of the supernatant were analysed by western blot.

### Western blotting

This was performed essentially as described previously^22^. The following secondary antibodies were used for western blot: goat anti-rabbit-coupled to horseradish peroxidase (HRP) and goat anti-mouse coupled to HRP (Biorad).

### Immunocytochemistry in tsA-201 cells

The method used is essentially as previously described^22^. Cells were transfected in low-serum medium. In co-transfection conditions, α_2_δ-1 and the stated TSP construct were used in a ratio of 1:1. For tsA-201 cells transfected separately, α_2_δ-1 was transfected directly onto poly-lysine coated coverslips and TSP4_HA-expressing cells from a different flask were layered onto α_2_δ-1-expressing cells 16–24 h after transfection. Cells were then incubated for a further 16–24 h before being fixed.

Cells were fixed with 4% paraformaldehyde in TBS for 5 min at room temperature, and then washed twice with TBS. Either no permeabilisation step was used, or cells were permeabilised for 15 min with 0.02% Triton X-100. The primary antibodies used were: mouse anti α_2_δ-1 (1:100) with rabbit anti-HA (1:500) for experiments where α_2_δ-1 and TSP4_HA were co-expressed; rabbit anti α_2_δ-1 (1:100) with mouse anti-Golgin-97 (1:500) or mouse anti-PDI (1:100) antibodies. Primary antibodies were incubated overnight at 4 °C, followed by the secondary antibodies, fluorescein isothiocyanate (FITC)-conjugated anti-mouse (1:500, Sigma) or anti-rabbit Alexa Fluor 594 (1:500, Invitrogen), for 1 h at room temperature. DAPI (4′,6-diamidine-2′-phenylindole dihydrochloride) was also used to visualise the nuclei. Cells were mounted in Vectashield (Vector laboratories, Burlingame, CA) to reduce photobleaching. Cells were examined on a confocal laser scanning microscope (Zeiss LSM780, except in Fig. 6A,B, taken with an LSM510), using a ×63 (1.4 NA) oil-immersion objective. Confocal optical sections were 1 μm. Photomultiplier settings were kept constant in each experiment and all images were scanned sequentially. Image processing was performed using ImageJ. Data illustrated are representative of images taken of at least 10 cells for non-permeabilised conditions and >100 cells for permeabilised conditions, from 3 independent experiments, unless otherwise stated.

## Additional Information

**How to cite this article**: Lana, B. *et al.* Thrombospondin-4 reduces binding affinity of [^3^H]-gabapentin to calcium-channel α_2_δ-1-subunit but does not interact with α_2_δ-1 on the cell-surface when co-expressed. *Sci. Rep.* **6**, 24531; doi: 10.1038/srep24531 (2016).

## Supplementary Material

Supplementary Information
