# Supplementary material for: Thrombospondin-4 reduces binding affinity of [3H]-gabapentin to calcium-channel α2δ-1-subunit but does not interact with α2δ-1 on the cell-surface when co-expressed
Source: Sci Rep. 2016 Apr 14;6:24531. doi: 10.1038/srep24531 (PMC4830977; doi:10.1038/srep24531)
Supplement: Supplementary Information [file srep24531-s1.pdf]

Thrombospondin-4 reduces binding affinity of [^{3}H]-gabapentin to calcium-channel $\alpha_2\delta$-1-subunit but does not interact with $\alpha_2\delta$-1 on the cell-surface when co-expressed

Beatrice Lana†, Karen M Page†, Ivan Kadurin, Shuxian Ho, Manuela Nieto-Rostro and Annette C Dolphin*

Supplementary data

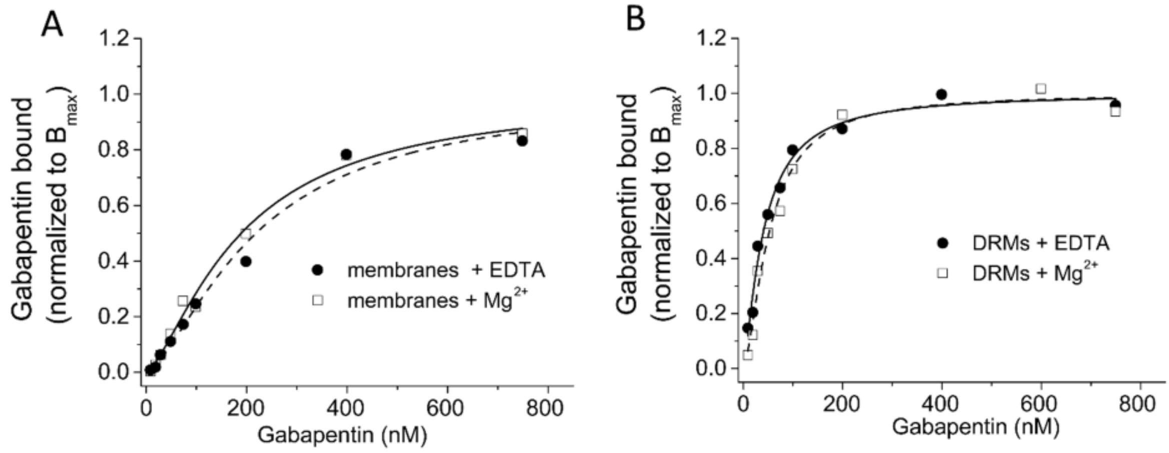

Supplementary Figure S1 Effect of Mg^{2+} on ^{3}H-gabapentin binding to $\alpha_2\delta$-1 in rat brain tissue.

Specific binding of ^{3}H-gabapentin was measured in a membrane preparation (A) and peak DRM fraction (B) of adult rat brain. The membranes and DRM fractions were prepared and the assay performed using EDTA 2mM (closed circles, fit with solid line) or 2 mM Mg^{2+} (open squares, fit with dashed line). The saturation binding curves are from one experiment, carried out in triplicate. Data were normalized to each mean B_{max} to illustrate the lack of difference in K_D values. (A) In brain membranes, the parameters for ^{3}H-gabapentin binding were: in the presence of Mg^{2+},K_D = 217.9 nM and n_H = 1.49; in the presence of EDTA, K_D = 189.1 nM, and n_H = 1.42. The respective B_{max} values were 0.63 pmol/mg protein in the presence of Mg^{2+} and 1.44 pmol/mg protein in the presence of EDTA. (B) In brain DRMs, the binding parameters for ^{3}H-gabapentin binding were: in the presence of Mg^{2+}, K_D = 52.7 nM, and n_H = 1.55; in the presence of EDTA, K_D = 40.9 nM and n_H = 1.34. The respective B_{max} values were 7.0 pmol/mg protein in the presence of Mg^{2+} and 12.2 pmol/mg protein in the presence of EDTA.

Supplementary Figure S2: Western blot analysis of co-immunoprecipitation of $\alpha_2\delta$-1 with TSP4

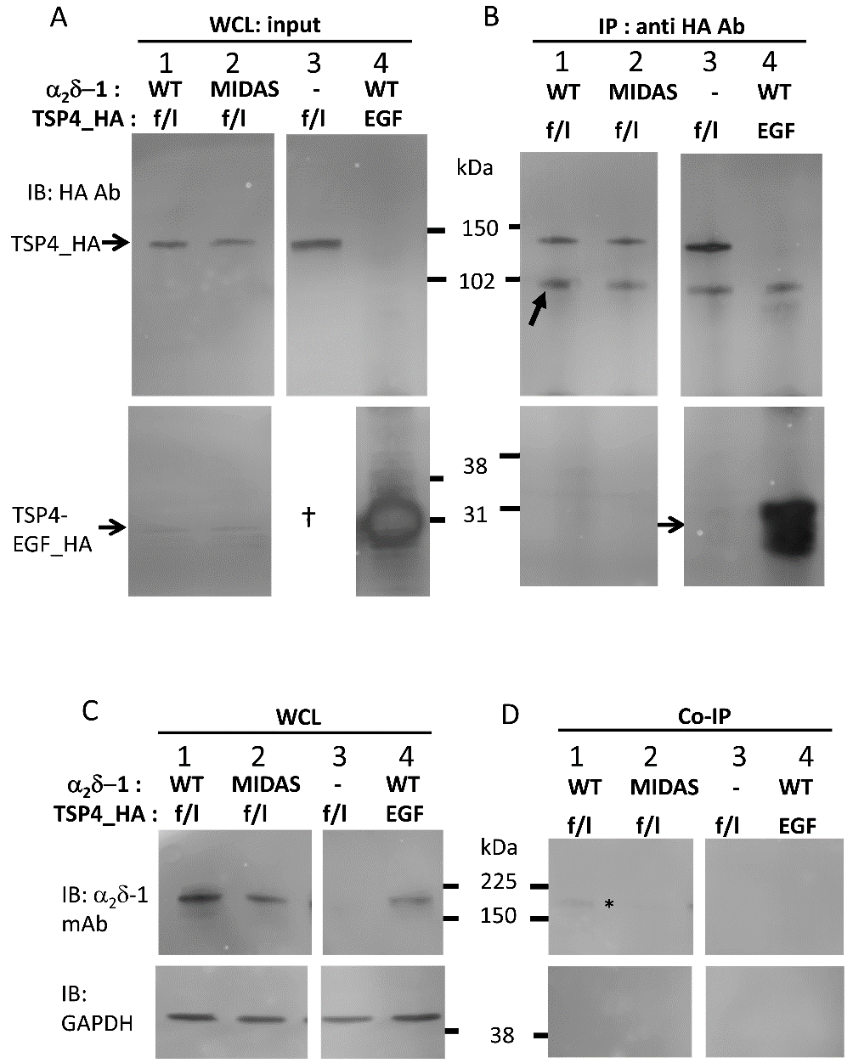

A, B: Immunoblots of WCL (A, 10 µg protein) and immunoprecipitation (B, 3 µl) of TSP4_HA with rabbit anti HA antibody from cells co-transfected with $\alpha_2\delta$-1 plus TSP4_HA (lane 1), $\alpha_2\delta$-1 MIDAS^{AAA} plus TSP4_HA (lane 2), TSP4_HA plus β1b instead of $\alpha_2\delta$-1 as a control (lane 3) and $\alpha_2\delta$-1 plus TSP4-EGF_HA (lane 4). All data are from the same blots, with irrelevant lanes removed. f/l = full-length. † absent due to technical error. White arrow, IgG band present in all lanes.

C, D: Immunoblots for $\alpha_2\delta$-1 of WCL (C, 10 µg protein) and co-immunoprecipitation (D, 20 µl) of $\alpha_2\delta$-1 (*) with TSP4_HA, using same material as in A, B. The bottom panels represent GAPDH loading and co-immunoprecipitation controls.

Supplementary Figure S3: Full length TSP4 binds to LRP1.

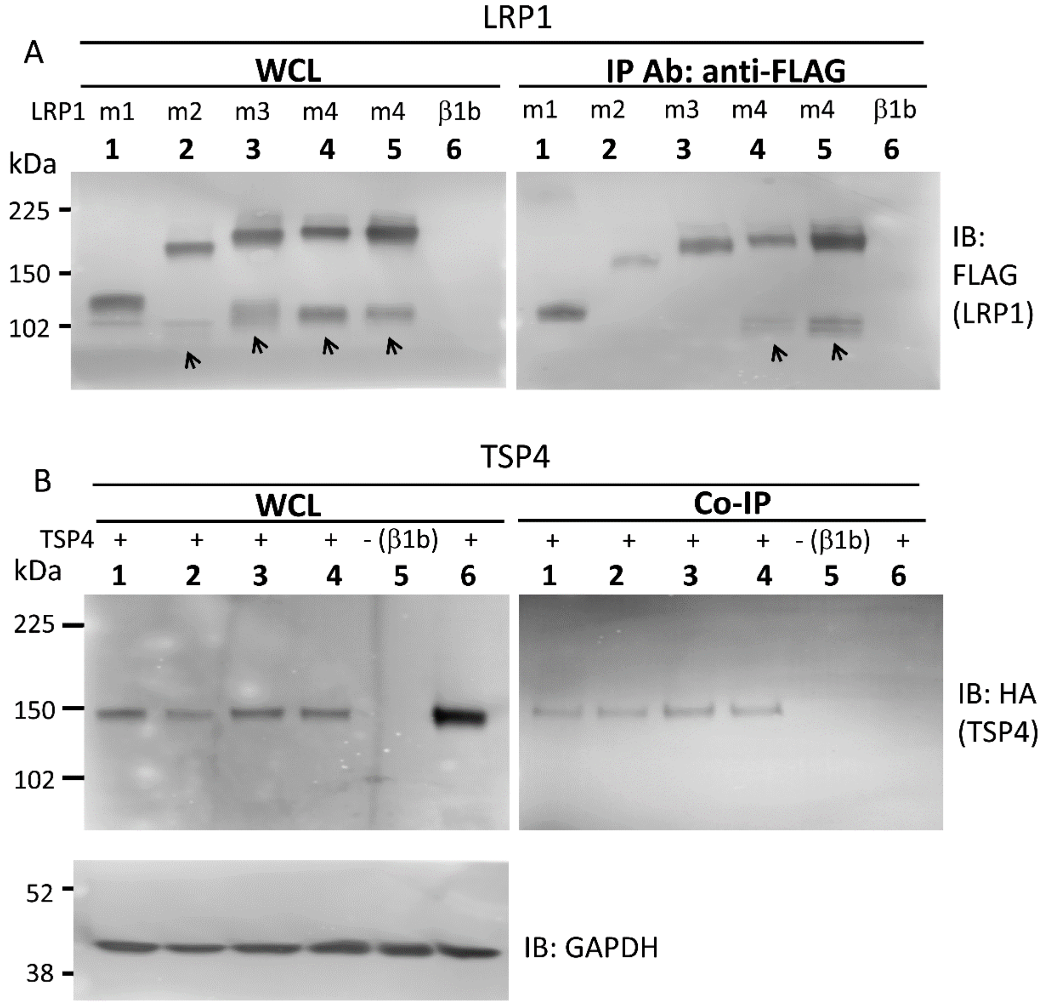

A: Western blot analysis of immunoprecipitation of LRP1 minigenes 1-4_FLAG, and co-immunoprecipitation of TSP4_HA (lanes 1 to 4). As negative controls, the intracellular calcium channel auxiliary subunit β1b was used in the transfection mix with LRP1 minigene 4, instead of TSP4_HA (lane 5) or β1b was co-transfected with TSP4_HA (lane 6). Top panel: LRP1 immunoblots with FLAG antibody for WCL (left) and immunoprecipitate (right). The arrowheads indicate the cleaved form of LRP1 minigenes 2-4, corresponding to the N-terminal ligand-binding domain subunits. Middle panel: TSP4_HA immunoblots using HA Ab, for WCL (left) and showing co-immunoprecipitated TSP4 (right). GADPH was used as loading control (bottom panel). 10 µg protein was loaded for WCL and 15 µl of immunoprecipitated material was loaded on the gels

Supplementary Table S1:

Quantification of TSP4-$\alpha_2\delta$-1 co-immunoprecipitation experiments

Transfected constructs	TSP4 ratio i.p /WCL	$\alpha_2\delta$-1 ratio i.p /WCL
$\alpha_2\delta$-1 TSP4_HA	1.50 ± 0.57 (8)	0.31 ± 0.12 (8)

The ratio of band intensity in immunoprecipitation/WCL is given for both TSP4 and $\alpha_2\delta$-1. The same amount of WCL protein and the same volume of immunoprecipitated material is loaded on the gels for detection of both TSP4 and $\alpha_2\delta$-1, or a correction for the amount loaded on the gel is applied to ensure correct comparison (see legends to Fig. 4 and Supplementary Fig. S2). The number of experiments is given in parentheses, and only those experiments showing co-immunoprecipitation are included.

Supplementary Table S2

Composition of Lysis and washing buffers used in co-immunoprecipitation assay

Lysis buffer, pH 8	50mM Tris, 150 mM NaCl, 1% Igepal, 0.5% Na deoxycholate, 0.1% SDS, 2 mM Mg^{2+}, EDTA-free protease inhibitor cocktail
High detergent washing buffer, pH 8: Used for the first 3 washes	20mM Tris, 150 mM NaCl, 1% Igepal, 2 mM Mg^{2+}, EDTA-free protease inhibitor cocktail
High salt washing buffer,pH 8: Used for second round of 3 washes	20 mM Tris, 0.5 M NaCl, 0.1 % Igepal, 2 mM Mg^{2+}, EDTA–free protease inhibitor cocktail
Low detergent and salt washing buffer, pH 8: Used for last round of 3 washes	20 mM Tris, 150mM NaCl, 0.1% Igepal, 2 mM Mg^{2+}, EDTA –free protease inhibitor cocktail

In the experiments shown in Figs. 4 and Fig. 5, 1 mM Mg^{2+}/1 mMCa^{2+} was used in place of 2 mM Mg^{2+}.
